# Deletion of Clock Gene *Period1* (*Per1*) in Neurons but Not in Astrocytes Shortens Clock Period and Diminishes Light-Mediated Rapid Phase Advances in Mice

**DOI:** 10.3390/clockssleep8010009

**Published:** 2026-02-23

**Authors:** Dan-Adrian Epuran, Urs Albrecht

**Affiliations:** Department of Biology, University of Fribourg, 1700 Fribourg, Switzerland; dan-adrian.epuran@unifr.ch

**Keywords:** light, resetting, phase shift, entrainment, advance, delay, *Per1* knock-out

## Abstract

The circadian clock enables organisms to anticipate daily recurring events and synchronize their internal rhythms with environmental cues, such as light, aligning with the day/night cycle. Central to the molecular mechanisms of the circadian clock and light sensing are the *Period* (*Per*) 1 and 2 genes. While the roles of *Per2* in astrocytes and neurons have been characterized, the specific contributions of *Per1* remain less understood. Previous research has shown that *Per2* in neurons, but not astrocytes, influences phase shifts, whereas the regulation of the circadian period involves *Per2* in both cell types. In this study, we investigated the role of *Per1* in neurons and astrocytes in modulating the circadian period and phase shifts. Using an Aschoff Type I protocol (constant darkness) combined with 15 min light pulses at circadian times (CT) 10, 14, and 22, we found that the absence of *Per1* in neurons—but not in astrocytes—significantly affected both the circadian period and phase advance shifts in response to light at CT22.

## 1. Introduction

The circadian system comprises organ- and tissue-specific clocks that regulate and synchronize physiological functions on a 24 h time scale, aligning them with the environmental day-night cycle. Disruption of this alignment—such as that caused by shift work or jet lag—impairs the regulation of bodily functions and may ultimately contribute to metabolic syndromes, addictive behaviors, cardiovascular disease, and neurological disorders [1].

At the cellular level, the circadian clock operates through a transcriptional-translational feedback loop with an approximately 24 h period. In mammals, the core clock genes include *Bmal1* and *Clock* (or its homolog *Npas2*), which activate the transcription of *Per* and *Cry* genes. These, in turn, inhibit their own activation, forming a negative feedback loop. The nuclear receptors REV-ERBα and RORα further regulate the expression of *Bmal1*, *Clock*, and *Npas2* by repressing or activating their transcription, respectively, thereby completing the core clock mechanism [2]. This mechanism can be modulated by: (1) molecules that bind to nuclear receptors—such as free fatty acids and glucocorticoids acting on PPARα and REV-ERBα—and (2) indirectly via glutamate and its receptors [3].

Circadian behavioral activity is primarily modulated by light. Changes in light intensity trigger adaptive responses, either advancing or delaying the organism’s activity phase, depending on the timing of exposure. For instance, a 15 min light pulse late in the subjective night (e.g., circadian time 22; CT22) advances the clock phase, whereas light exposure early in the subjective night (e.g., CT14) delays it [4,5,6]. At the cellular level, light stimulates the release of neurotransmitters—such as glutamate—at synapses connected to the suprachiasmatic nuclei (SCN), the master pacemaker of the circadian system. This leads to the induction of immediate early genes, such as *Fos*, and clock genes, such as *Per1* and *Per2* [7,8,9,10,11].

Notably, mutation of the *Per2* gene in all cells of mice shortens the circadian period, diminishes phase delays, and enhances phase advances. In contrast, global deletion of *Per1* shortens the period and reduces phase advances only [12,13]. Cell-type-specific deletion of *Per2* in astrocytes or neurons also shortens the clock period, but only neuronal deletion affects phase shifts [14]. The distinct role of *Per1* in astrocytes and neurons regarding period regulation and phase-shifting remains unclear. Therefore, we investigated the contributions of *Per1* in these cell types to the circadian period and light-induced phase-shifting responses. To this end, we analyzed the wheel-running activity of mice with deletion of *Per1* in astrocytes (*Per1*GKo) or neurons (*Per1*NKo) before and after a brief light pulse of 15 min under constant dark conditions (DD) and compared them to controls (NCo and GCo, respectively). We find that deletion of *Per1* in neurons, but not in astrocytes, shortens clock period and diminishes light-mediated rapid phase advances. However, it remains elusive how *Per1* in neurons regulates phase advances.

## 2. Results

### 2.1. Per1 Is Absent in Neuronal or Astrocytic Knock-Out Mice

To investigate the cell-type-specific role of *Per1* in regulating circadian period and light responsiveness, we generated astrocytic-specific (*Per1*G; *Per1*/*Gfap-Cre*) and neuron-specific (*Per1*N; *Per1*/*Nes-Cre*) *Per1* knock-out (KO) mice. Mice carrying a floxed *Per1* allele [15] (EMMA: 14846) were crossed with transgenic mice expressing *Cre*-recombinase under the control of either the *gfap*-promoter (*Gfap-Cre*, JAX: 004600) or the *nestin*-promoter (*Nes-Cre*, EMMA: EM04561).

**Figure 1:**
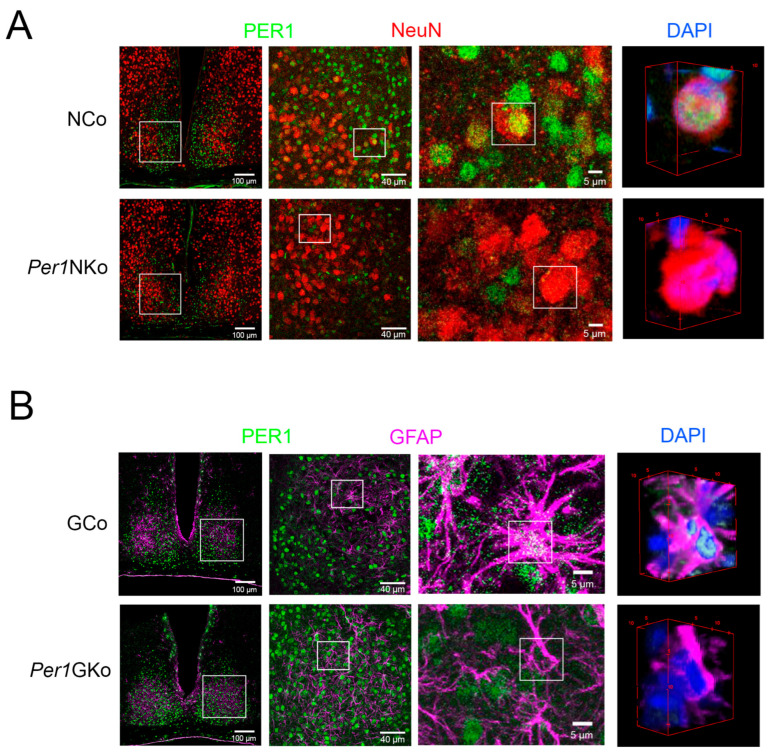
Immunohistochemistry of PER1 expression in the suprachiasmatic nuclei (SCN) of neuronal control (NCo), neuronal *Per1* knock-out (*Per1*NKo), astrocytic control (GCo) and glial *Per1* knock-out (*Per1*GKo) mice at Zeitgeber time (ZT) 12. (**A**) Photomicrographs of SCNs from NCo and *Per1*NKO mice at low to high magnification (from left to right, white line = 100 µm, 40 µm, 5 µm). White squares indicate the magnified areas. The images on the right show 3D reconstructions of cells with DAPI nuclear staining (blue). Green = PER1, red = NeuN specific to neuronal cells. (**B**) Photomicrographs of SCNs from GCo and *Per1*GKO mice at low to high magnification (from left to right, white line = 50 µm, 10 µm, 5 µm). White squares indicate the magnified areas. The images at right show 3D reconstructions of cells with DAPI nuclear staining (blue). Green = PER1, pink = glial fibrillary acidic protein (GFAP) specific to astrocytes and some other glial cells, including radial glia, Müller glia, ependymal cells, peripheral glial cells, and myoepithelial cells [16].

To confirm cell-type-specific deletion of *Per1* in neurons and astrocytes, we performed immunohistochemistry on suprachiasmatic nucleus (SCN) tissue harvested at ZT12 using PER1-specific antibodies (Figure 1, green). We selected the SCN to confirm our deletion due to its high expression of the PER1 protein, particularly at ZT12 [11]. Neurons were labeled with an anti-NeuN antibody (red) (Figure 1A). In neuronal control mice (NCo; *Nes-Cre*), PER1 and NeuN co-localized, producing a yellow signal (Figure 1A, upper row, white square at 5 µm). A 3D reconstruction of the neuron at 5 µm confirmed co-expression of PER1, NeuN, and the nuclear stain DAPI (blue), resulting in a yellow composite signal. In *Per1*NKo mice, PER1 expression was absent in neurons (Figure 1A, lower row), indicating successful deletion of *Per1* in this cell type.

Astrocytes were labeled with an anti-GFAP antibody (Figure 1B, magenta). In astrocytic control mice (GCo; *Gfap-Cre*), PER1 and GFAP co-expressed, producing a white signal (Figure 1B, upper row, white square at 5 µm). A 3D reconstruction of the astrocyte at 5 µm showed co-expression of PER1, GFAP, and DAPI (blue), resulting in a white/cyan signal. In *Per1*GKo mice, PER1 expression was absent in astrocytes (Figure 1B, lower row), confirming successful deletion of *Per1* in astrocytic cells.

### 2.2. Lack of Per1 Expression in Neuronal or Astrocytic Knock-Out Mice Does Not Abolish Circadian Rhythmicity but Slightly Reduces Phase Advances in Per1NKo Animals

**Figure 2:**
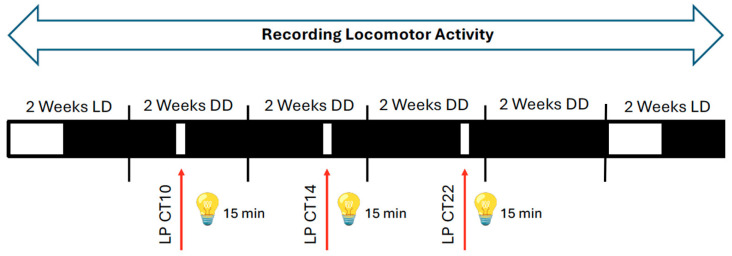
Experimental design for application of 15 min light pulses (LPs, 1000 LUX, red arrows) using the Aschoff type I protocol. Mice were kept in a light/dark cycle (LD) for 2 weeks, and then they were released into constant darkness (DD) before receiving an LP at circadian time (CT) 10. Subsequently, the animals were kept for 2 weeks in DD before they received an LP at CT14 (early subjective night). The mice were again kept for 2 weeks in DD, and then they received an LP at CT22 (late subjective night). Subsequently, the animals were kept again in DD for 2 weeks. During the whole experiment, locomotor activity was recorded. White and black bars represent light or dark, respectively.

**Figure 3:**
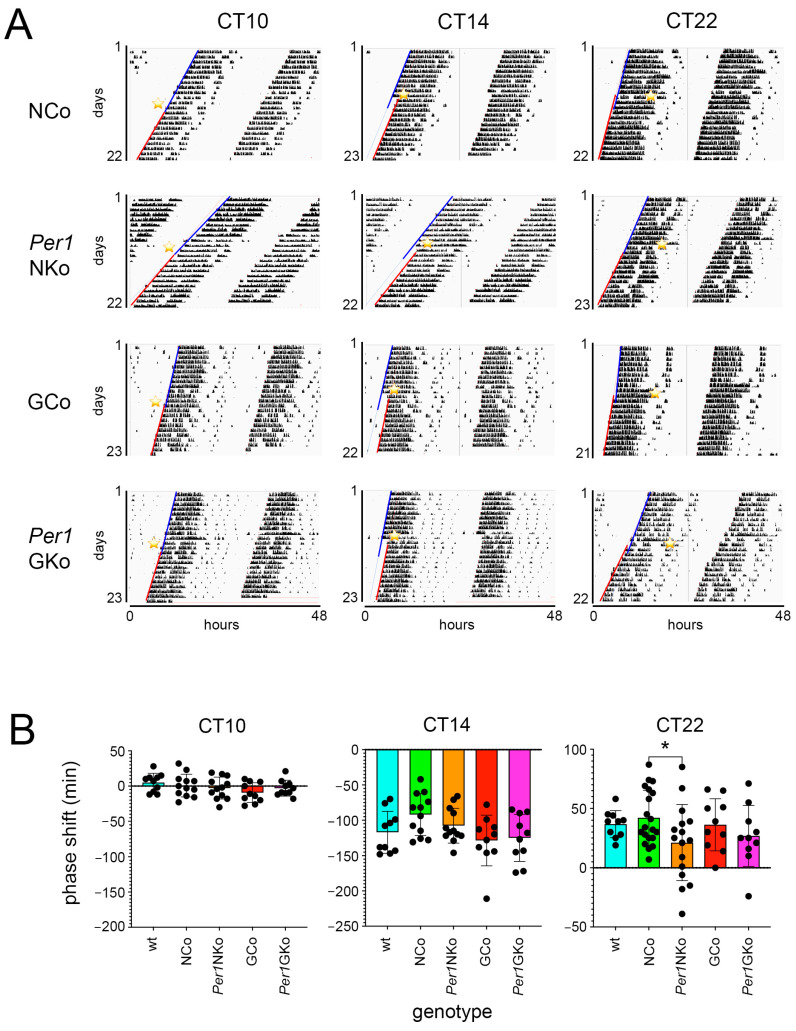
Wheel-running activity and effect of light pulses on control and cell-type-specific *Per1* knock-out mice under constant darkness (DD) conditions (Aschoff type I protocol). Independent measures throughout light pulse conditions of both male and female C57BL/6 mice across all genotypes. (**A**) Representative actograms of wheel-running activity of neuronal control (NCo), neuronal *Per1* knock-out (*Per1*NKo), astrocytic control (GCo) and astrocytic *Per1* knock-out (*Per1*GKo) animals. A 15 min light pulse (LP) was applied at circadian times (CTs) 10, 14, and 22 (yellow asterisk). The blue lines indicate onset of wheel-running activity before the LP and the red lines onset of activity after the LP. (**B**) Quantification of the distances between the blue and red lines in A represents the amount of phase shift in minutes after an LP at CT10 (left panel), CT14 (middle panel) and CT22 (right panel). Data are represented as mean ± SEM with *n* = 10 for wt (light blue), GCo (red), *Per1*GKo (pink) for LPs CT10, CT14 and CT22, and *n* = 12 for NCo (green), *Per1*NKo (orange), for LP at CT10 and CT14. A diminished phase advance in *Per1* NKo mice was observed in response to an LP at CT22 (*n* = 20 for NCo and 16 for Per1NKO, * *p* = 0.0283, t = 2.523, DF = 61, Šídák’s multiple comparisons test) (Table A1, Table A2 and Table A3).

To assess the impact of light on mice lacking *Per1* in neurons or astrocytes, we employed an Aschoff Type I protocol to evaluate light-induced rapid phase shifts under constant darkness [17,18]. The experimental design is illustrated in Figure 2 (see also Section 4). All mouse strains were housed in constant darkness (DD) with access to a running wheel, and locomotor activity was recorded and visualized as double-plotted actograms (Figure 3A).

After approximately 10 days in DD, animals received a 15 min light pulse (LP) at circadian time (CT) 10, CT14, and CT22 (Figure 3A, yellow stars). The LPs were selected according to the phase response curve described in [4]. An LP at CT10 was selected as a control because this time point is in the dead zone of the phase response curve (PRC) and therefore should not induce a phase shift response. CT14 and CT22 were chosen because they represent strong phase delay or advance responses of the PRC, respectively. Following the LP, mice remained in DD, and activity onsets were marked with lines—blue for pre-LP and red for post-LP. The shift between blue and red lines was used to quantify phase delays or advances in activity onset.

Quantitative analysis revealed no significant effect of the LP at CT10 across all strains (Figure 3B, left panel). The LP at CT14 induced phase delays that were consistent among all groups (Figure 3B, middle panel). However, the LP at CT22 resulted in phase advances in all strains, with the exception of *Per1*NKo mice, which exhibited significantly reduced phase advances compared to NCo controls (Figure 3B, right panel).

These findings suggest that *Per1* expression in neurons—but not in astrocytes—is involved in mediating light-induced phase advances in circadian behavior.

### 2.3. Period Is Shortened in Mice Lacking Per1 in Neurons, and Light at CT22 Shortens Period Across Each Strain

**Figure 4:**
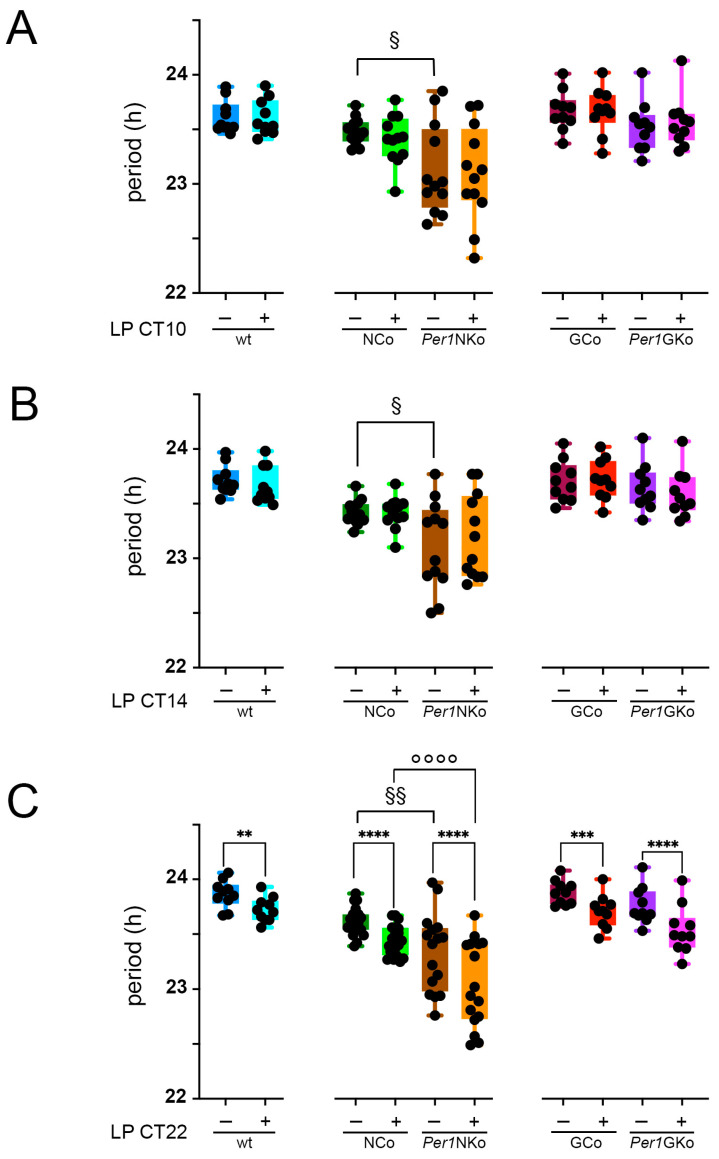
Period length in control (wt, NCo, GCo) and *Per1* knock-out animals (*Per1*NKo, *Per1*GKo) and the effect of a light pulse (LP) on period. Independent measures throughout light pulse conditions of both male and female C57BL/6 mice across all genotypes. (**A**) An LP at CT10 does not affect the period length across all genotypes; however, deletion of *Per1* in the neurons leads to a shorter period. Data are represented as mean ± SEM with *n* = 10 for wt, GCo, *Per1*GKo and *n* = 12 for NCo, *Per1*NKo. Period in *Per1*NKo mice (brown) before the LP is significantly shorter compared to NCo animals (dark green) (^§^ *p* = 0.0213, t = 2.665, DF = 44, *n* = 12, Šídák’s multiple comparisons test). (**B**) An LP at CT14 does not affect the period length across all genotypes; however, deletion of *Per1* in the neurons leads to a shorter period. Data are represented as mean ± SEM with *n* = 10 for wt, GCo, *Per1*GKo and *n* = 12 for NCo, *Per1*NKO. Period in *Per1*NKo mice (brown) before the LP is significantly shorter compared to NCo animals (dark green) (^§^ *p* = 0.0284, t = 2.551, DF = 44, *n* = 12, Šídák’s multiple comparisons test). (**C**) Period before and after an LP at CT22 are significantly different in all genotypes investigated (** *p* = 0.0025, t = 4.154, DF = 9, *n* = 10 for wt, paired *t*-tests **** *p* < 0.0001, t = 4.692, DF = 34, *n* = 20 for NCo, **** *p* < 0.0001, t = 6.9464, DF = 34, *n* = 16 for *Per1*NKo; Uncorrected Fisher’s LSD; *** *p* = 0.0006, t = 4.473, DF = 18, *n* = 10 for GCo and **** *p* < 0.0001, t = 5.689, DF = 18, *n* = 10 for *Per1*GKo, Šídák’s multiple comparisons test). Data are represented as mean ± SEM. Period in *Per1*NKo mice (brown) before the LP is significantly shorter compared to NCo animals (dark green) (^§§^ *p* = 0.0028, t = 3.098, DF = 68, *n* = 16–20, Uncorrected Fisher’s LSD). Also, after an LP, the CT22 period in *Per1*NKo mice (orange) is significantly shorter compared to NCo animals (green) (°°°° *p* < 0.0001, t = 4.382, DF = 68, *n* = 16–20, Uncorrected Fisher’s LSD) (Table A4, Table A5 and Table A6).

Using actograms derived from wheel-running activity recordings, we assessed the circadian period across all mouse strains. Notably, only mice lacking *Per1* in neurons (*Per1*NKo) exhibited a significantly shorter circadian period compared to their neuronal controls (NCo) under baseline conditions without LP (Figure 4A–C, § symbols, *p* = 0.0166, F (1,22) = 6.724, *p* = 0.0337, F (1,22) = 5.128, *p* = 0.0004, F (1,34) = 15.49). This finding aligns with previous reports of reduced phase advances in global *Per1* knock-out mice [12,13].

We next examined whether light pulses at different circadian times affected the period. An LP at CT10 had no significant impact on the period across all genotypes (Figure 4A). Similarly, an LP at CT14 did not alter the period in all strains (Figure 4B).

Interestingly, an LP at CT22 resulted in a significant shortening of the circadian period in each genotype examined (Figure 4C, * symbols, *p* = 0.0025, WT mice, *p* < 0.0001, F (1,34) = 69.2, NCo and *Per1*NKo, *p* < 0.0001, F (1,18) = 51.63, Gco and *Per1*GKo). This suggests that a light-sensitive signaling pathway is activated at CT22 and remains functional regardless of *Per1* deletion in either neurons or astrocytes. Furthermore, we observed that period shortening due to the LP at CT22 is larger in *Per1*NKo compared to NCo controls (Figure 4C, °°°° symbol, *p* < 0.0001, F (1,34) = 69.2). This indicates that the neuronal cre-driver line does not account for the shortening of the period in *Per1*NKo mice.

### 2.4. Amplitude and Relative Power of Phase Are Reduced in Per1NKo Mice

**Figure 5:**
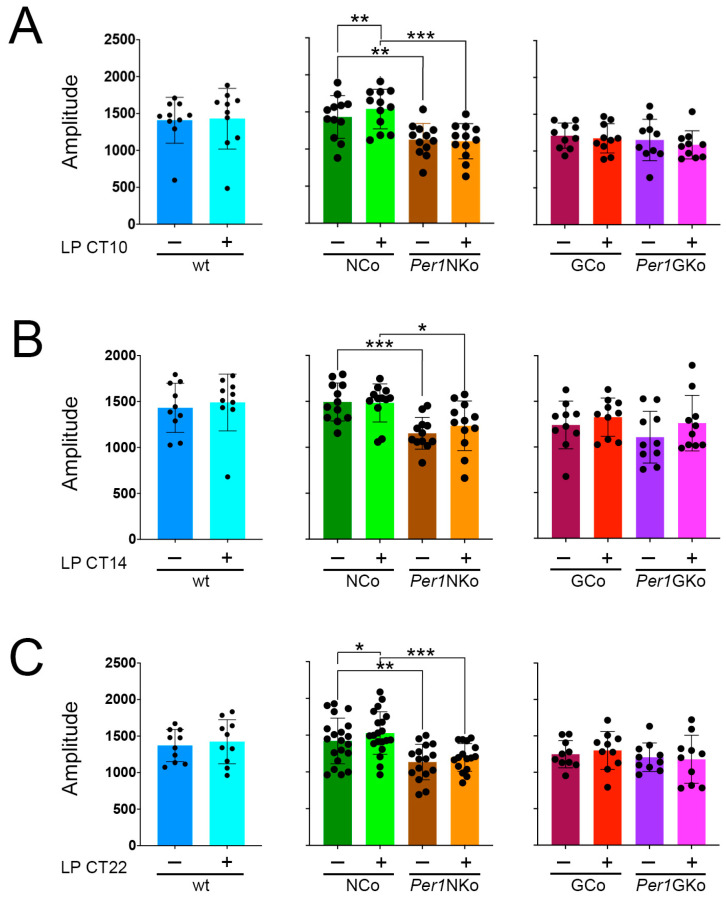
Amplitude in control (wt, NCo, GCo) and *Per1* knock-out animals (*Per1*NKo, *Per1*GKo) and effect of a light pulse (LP) on amplitude. Independent measures throughout light pulse conditions, of both male and female C57BL/6 mice across all genotypes. (**A**) An LP at CT10 only affects the amplitude in NCo mice, while *Per1*NKO mice show lower amplitudes compared to NCo mice. Data are represented as mean ± SEM with *n* = 10 for wt, GCo, *Per1*GKo and *n* = 12 for NCo, *Per1*NKo. Amplitude in the NCo mice changes following the LP at CT10 (** *p* = 0.0041, t = 3.201, DF = 22, Uncorrected Fisher’s LSD). Amplitude in *Per1*NKo mice before (brown) and after (orange) the LP is significantly lower compared to NCo animals (green) (** *p* = 0.0051, t = 2.947, DF = 44, *n* = 12, for before the LP CT10 and, *** *p* = 0.0001, t = 4.181, DF = 44, *n* = 12, for after the LP CT10, Uncorrected Fisher’s LSD). (**B**) An LP at CT14 only affects the amplitude in the *Per1*GKO mice, while the *Per1*NKO mice show lower amplitudes compared to the Nco mice. Data are represented as mean ± SEM with *n* = 10 for wt, GCo, *Per1*GKo and *n* = 12 for NCo, *Per1*NKo. Amplitude in *Per1*NKo mice before (brown) and after (orange) the LP is significantly lower compared to NCo animals (green) (*** *p* = 0.0008, t = 3.845, DF = 44, *n* = 12, for before the LP CT14 and, * *p* = 0.0148, t = 2.806, DF0 44, *n* = 12, Šídák’s multiple comparisons test). (**C**) An LP at CT22 only affects the amplitude in the NCo mice, while the *Per1*NKO mice show lower amplitudes compared to the NCo mice. Data are represented as mean ± SEM with *n* = 10 for wt, GCo, *Per1*GKo and *n* = 20 for NCo, *n* = 16 for *Per1*NKo. Amplitude in the NCo mice changes following the LP at CT22 (* *p* = 0.0168, t = 2.515, DF = 34, Uncorrected Fisher’s LSD). Amplitude in *Per1*NKo mice before (brown) and after (orange) the LP is significantly lower compared to NCo animals (green) (** *p* = 0.0020, t = 3.211, DF = 68, *n* = 16–20, for before the LP CT22 and, *** *p* = 0.0004, t = 3.709, DF = 68, *n* = 16–20, for after the LP CT22, Uncorrected Fisher’s LSD) (Table A7, Table A8, Table A9, Table A10, Table A11, Table A12, Table A13, Table A14 and Table A15).

We assessed circadian amplitude in all genotypes and found that light pulses (LPs) administered at CT10, CT14, and CT22 did not significantly alter amplitude in wt, *Per1*NKo, GCo and *Per1*GKo (Figure 5A–C). In NCo animals, a change in amplitude was observed at CT10 and CT22 (*p* = 0.0129, F (1,22) = 7.318, *p* = 0.0118, F (1,34) = 7.081) (Figure 5A,C). However, *Per1*NKo mice exhibited a markedly reduced amplitude compared to their neuronal controls (Figure 5A–C, middle panels, brown and orange columns, *p* = 0.0014, F (1,22) = 13.4, *p* = 0.0013, F (1,22) = 13.58, *p* = 0.0007, F (1,34) = 13,74) suggesting a potential weakening of the circadian oscillator in the absence of neuronal *Per1*.

**Figure 6:**
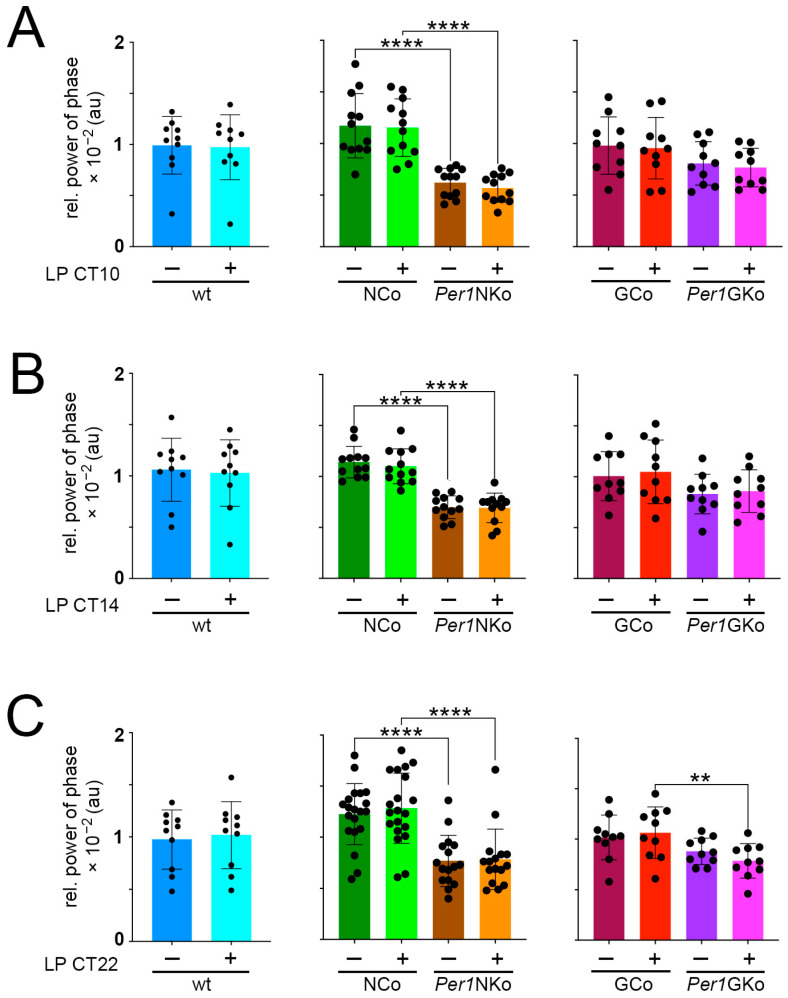
Relative power of phase in control (wt, NCo, GCo) and *Per1* knock-out animals (*Per1*NKo, *Per1*GKo) and effect of a light pulse (LP) on relative power of phase. Independent measures throughout light pulse conditions of both male and female C57BL/6 mice across all genotypes. (**A**) An LP at CT10 does not affect the relative power of phase across all genotypes, while the *Per1*NKO mice show a lower relative power of phase compared to the NCo mice. Data are represented as mean ± SEM with *n* = 10 for wt, GCo, *Per1*GKo and *n* = 12 for NCo, *Per1*NKo. Relative power of phase in *Per1*NKo mice before (brown) and after (orange) the LP is significantly lower compared to NCo animals (green) (**** *p* < 0.0001, t = 5.850, DF = 44, *n* = 12, for before the LP CT10 and, **** *p* < 0.0001, t = 6.213, DF = 44, *n* = 12, for after the LP CT10, Šídák’s multiple comparisons test). (**B**) An LP at CT14 does not affect the relative power of phase across all genotypes, while the *Per1*NKO mice show a lower relative power of phase compared to the NCo mice. Data are represented as mean ± SEM with *n* = 10 for wt, GCo, *Per1*GKo and *n* = 12 for NCo, *Per1*NKo. Relative power of phase in *Per1*NKo mice before (brown) and after (orange) the LP is significantly lower compared to NCo animals (green) (**** *p* < 0.0001, t = 7.363, DF = 44, *n* = 12, for before the LP CT14 and, **** *p* < 0.0001, t = 6.782, DF = 44, *n* = 12, for after the LP CT14, Šídák’s multiple comparisons test). (**C**) An LP at CT22 does not affect the relative power of phase across all genotypes, while the *Per1*NKO mice show a lower relative power of phase compared to the NCo mice. Only the *Per1*GKo with LP group shows a lower relative power of phase compared to its GCo with LP. Data are represented as mean ± SEM with *n* = 10 for wt, GCo, *Per1*GKo and *n* = 20 for NCo, *n* = 16 for *Per1*NKo. Relative power of phase in *Per1*NKo mice before (brown) and after (orange) the LP is significantly lower compared to NCo animals (green) (**** *p* < 0.0001, t = 4.704, *n* = 16–20, for before the LP CT22 and, **** *p* < 0.0001, t = 5.476, *n* = 16–20, for after the LP CT22, Šídák’s multiple comparisons test). Relative power of phase in *Per1*GKo (magenta) animals is lower compared to GCo animals (red) after an LP at CT22 (** *p* = 0.0075, t = 3.099, DF = 36, *n* = 10, Šídák’s multiple comparisons test) (Table A16, Table A17, Table A18, Table A19, Table A20, Table A21, Table A22, Table A23 and Table A24).

To further investigate oscillator strength, we quantified the relative power of phase in each genotype (Figure 6). An LP at CT10, CT14 and CT22 does not affect power of phase within each genotype. Consistent with the observed shorter period and reduced amplitude, *Per1*NKo mice showed significantly lower relative power of phase compared to controls either before or after an LP (Figure 6A–C, middle panels, *p* < 0.0001, F (1,22) = 37.91, *p* < 0.0001, F (1,22) = 61.72, *p* < 0.0001, F (1,34 = 37.33). Interestingly, power of phase was affected in *Per1*GKo compared to GCo controls after an LP at CT22 (Figure 6C, right panel, *p* = 0.0190, F (1,18) = 6.635).

Taken together, these observations indicate that *Per1*NKo mice have a weakened oscillator.

**Figure 7:**
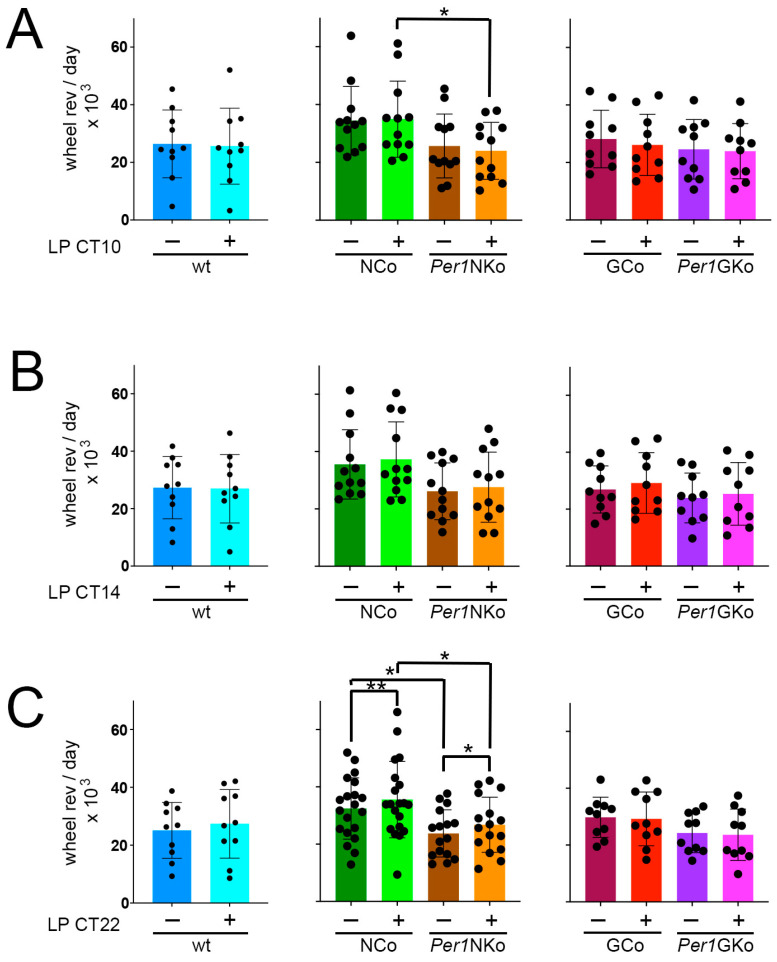
Total wheel-running activity in control (wt, NCo, GCo) and *Per1* knock-out animals (*Per1*NKo, *Per1*GKo) and effect of a light pulse (LP) on wheel-running activity. Independent measures throughout light pulse conditions of both male and female C57BL/6 mice across all genotypes. (**A**) *Per1*KO mice show lower total wheel-running activity compared to the NCo mice after an LP at CT10. Data are represented as mean ± SEM with *n* = 10 for wt, GCo, *Per1*GKo and *n* = 12 for NCo, *Per1*NKo. Total wheel-running activity in the *Per1*NKo mice is lower following the LP at CT10 (* *p* = 0.0489, t = 2.324, DF = 44, *n* = 12, Šídák’s multiple comparisons test). (**B**) An LP at CT14 does not affect total wheel-running activity across all genotypes, with no changes being observed between the *Per1*KO mice and their controls. Data are represented as mean ± SEM with *n* = 10 for wt, GCo, *Per1*GKo and *n* = 12 for NCo, *Per1*NKo. (**C**) An LP at CT22 affects the total wheel-running activity in the NCo and *Per1*NKo mice, but not in *Per1*GKO mice. Data are represented as mean ± SEM with *n* = 10 for wt, GCo, *Per1*GKo and *n* = 20 for NCo, *n* = 16 for *Per1*NKo. Total wheel-running activity in the NCo mice changes following the LP at CT22 (** *p* = 0.0061, t = 2.925, DF = 34, *n* = 20, Uncorrected Fisher’s LSD) and in the *Per1*NKo (* *p* = 0.0157, t = 2.544, DF = 34, *n* = 16, Uncorrected Fisher’s LSD). Total wheel-running activity in *Per1*NKo mice before (brown) and after (orange) the LP is significantly lower compared to NCo animals (green) (* *p* = 0.0193, t = 2.397, DF = 68, *n* = 16–20, for before the LP CT22 and, * *p* = 0.0182, t = 2.421, DF = 68, *n* = 16–20, for after the LP CT22, Uncorrected Fisher’s LSD) (Table A25, Table A26, Table A27, Table A28, Table A29, Table A30, Table A31, Table A32 and Table A33).

Overall wheel-running activity indicated no differences between the knock-out lines and their respective controls except for *Per1*NKo mice compared to their NCo (Figure 7A, C, middle panels, *p* = 0.0441, F (1,22) = 4.558, *p* =0.0187, F (1,34) = 6.097). Sometimes an LP affected total activity, as observed for NCo and *Per1*NKo (Figure 7C, middle panel, *p* = 0.0005, F (1,34) = 14.79). These observations indicate that the seen effects may influence some of the other circadian parameters; however, total activity probably has a marginal effect on phase shifts.

## 3. Discussion

In this study, we investigated how the absence of the *Per1* gene in neurons and astrocytes affects the circadian period and phase, using wheel-running activity as a behavioral paradigm. Our experiments revealed that deletion of *Per1* in astrocytes had no or a weak impact on these parameters. In contrast, the absence of *Per1* in neurons led to a shortened circadian period and diminished phase advances in response to a light pulse administered at CT22.

Previous studies have reported that *Per1::luc* reporter expression is undetectable in glial cells within organotypic SCN slice cultures from a rat brain [19]. However, *Per1::luc* rhythms were observed in cultured astrocytes derived from cortical rat tissue [20]. This discrepancy suggests that *Per1* expression in astrocytes may be substantially lower or more diffused than in neurons, where *Per1::luc* expression is readily detectable [19]. Our immunohistochemistry data support this interpretation (Figure 1): We observed higher expression of PER1 protein in SCN neurons (Figure 1A), which was markedly lower in astrocytes (Figure 1B).

Consistent with these findings, most behavioral circadian parameters assessed in mice lacking *Per1* in astrocytic cells (*Per1*GKo) were similar to those of astrocytic controls. Phase shifting remained unaffected (Figure 3), period was comparable (Figure 4), amplitude was normal (Figure 5), and relative power of phase was unchanged, with the exception of after the LP at CT22 (Figure 6). Total wheel-running activity also matched that of control animals (Figure 7). These results suggest that astrocytic *Per1* plays a minimal, if any, role in regulating behavioral circadian clock parameters in vivo.

In contrast, deletion of *Per1* in neurons resulted in reduced light-induced phase advances (Figure 3), shortened period (Figure 4), and decreased amplitude (Figure 5), culminating in reduced relative power of phase (Figure 6)—indicative of a weakened circadian oscillator. Notably, these changes were most likely not attributable to differences in total wheel-running activity, although differences in animals receiving an LP at CT22 were observed (Figure 7, middle panel). These findings are in line with previous reports [19] and reinforce the notion that *Per1* primarily influences behavioral circadian parameters through neuronal mechanisms rather than astrocytic ones.

Several studies have demonstrated that *Per1* expression in the SCN can be induced by brief light exposure [7,10,11]. Moreover, global deletion of *Per1* in mice has been shown to impair phase advances without affecting phase delays [12], suggesting that light-induced *Per1* expression is specifically linked to phase advances. Our findings support and extend this view by showing that *Per1* expression in neurons—but not astrocytes—is important for light-induced phase advances (Figure 3).

The reduction in phase advances observed in *Per1*NKo mice cannot be solely attributed to light-induced period shortening at CT22 (Figure 4C). All genotypes exhibited period shortening following a light pulse at CT22, which could be interpreted as an earlier onset of activity the next day, contributing to the phase advances seen in Figure 3. This effect was also present in *Per1*NKo mice (Figure 4C, orange bar), and the difference in period between NCo and *Per1*NKo mice increased post-light pulse, with *Per1*NKo mice showing an even shorter period (Figure 4C, §§ and °°°°). If period shortening alone accounted for phase advances, *Per1*NKo mice would be expected to show greater advances than controls. However, the opposite was observed, indicating that *Per1*’s role in phase advancement in response to light at CT22 involves a molecular pathway distinct from its role in period regulation.

The functions of *Per1* and *Per2* genes appear to be distinct [12,21,22], and this distinction is evident when comparing their roles in neurons and astrocytes. In this study, we found that *Per1* in neurons—but not astrocytes—was critical for regulating period and phase advances. In contrast, *Per2* influenced period in both cell types, but only neuronal *Per2* was essential for phase delays [14]. This functional segregation aligns with previous reports showing that *Per1*-reporter expression is predominantly neuronal, while *Per2*-reporter expression is more restricted to non-neuronal populations [23]. Our findings also corroborate a recent study indicating that astrocytes regulate circadian period but not phase in the SCN [24].

There are several limitations to our study that should be acknowledged. First, assessing the presence or absence of *Per1* in astrocytes is challenging. Techniques such as Western blotting and in situ hybridization lack the sensitivity required to selectively measure astrocytic *Per1* levels. While PCR analysis following FACS sorting of astrocytes would provide more reliable data, this approach would require a large number of animals, making it impractical. Consequently, we relied on immunohistochemistry (IHC), which, although not quantitative, offers a reasonable indication of *Per1* levels in astrocytes. Given this limitation, conclusions regarding the absence of *Per1* in astrocytes should be interpreted with caution. Second, we cannot exclude the possibility that light-induced effects on the circadian period contribute to the observed phase advances.

Although the *Per1* and *Per2* genes function within the same molecular autoregulatory feedback loop, this study suggests that their relative contributions may vary depending on the cell type. Such variation enables differential regulation of cell-type-specific output mechanisms, as well as cell-type-dependent responses, to clock-related sensory input. This idea is supported by findings that *Per1* and *Per2* control distinct sets of clock-regulated genes [25], thereby exerting differential effects on behavior [12,21]. More recent work has demonstrated that functional partitioning can occur at the level of cell-type-specific oscillators [26]. Taken together, these insights highlight how cell-type-specific deletions of *Per* genes can be leveraged to address fundamental questions in circadian biology (e.g., intercellular coupling, cell-autonomous oscillations) and to explore the role of circadian timing in processes such as cell proliferation, sleep, and the development of neurological disorders, including Alzheimer’s disease.

In summary, this study shows that astrocytic *Per1* appears to have limited importance for circadian regulation, whereas astrocytic *Per2* contributes to period control but not to light-mediated phase shifts. In neurons, both *Per* paralogs are essential: *Per1* for phase advances and *Per2* for phase delays.

## 4. Materials and Methods

### 4.1. Housing of Mice and Mouse Strains

Male and female mice (50:50), aged 2–6 months, on a C57Bl/6 background, were placed in isolated light cabinets as previously described in Jud et al., 2005 [27]. Entrainment was done in a constant 12:12 h light/dark cycle. Temperature (22 ± 2 °C monitored by temperature sensor, Technoline WS-9410, Berlin, Germany), humidity (40–50% monitored by humidity sensor, Technoline WS-9410, Berlin, Germany), and illumination (1000 Lux monitored by Luxmeter, Testo, GmbH and Co., Titisee-Neustadt, Germany) were kept constant in all cabinets. Each mouse was housed individually in cages (L: 280 mm × W: 105 mm × H: 125 mm) containing a running wheel made of steel (115 mm in diameter, Trixie GmbH, Tarp, Germany). All mice were provided with sufficient woodchip bedding, so as not to block the running wheel, and enrichment materials such as: red-square house, a piece of carton, neslet (5 × 5 cm) and an open-sided tube. Food and water were provided ad libitum.

The neuronal-specific *Per1*NKo (*Per1* fl/fl × *Nestin-cre*) and astrocytic-specific *Per1*GKo (*Per1 fl/fl* × *Gfap-cre*) were obtained by breeding the *Per1 fl/fl* mice described in Olejniczak et al., 2021; EMMA: 14846 [15] against the *Nestin-cre* mice [28] (EMMA: EM04561) and *Gfap-cre* mice [29] (JAX: 004600). Recombination was verified by RT-PCR analysis, and animals were backcrossed 6 times. However, to exclude contributions from the cre-driver lines, we used them as additional controls to wild-type animals. All experiments and procedures were performed according to the Schweizer Tierschutzgesetz guidelines and approved by the Canton of Fribourg and the cantonal commission for animal experiments (2021-17-FR, 33789).

### 4.2. Monitoring of Circadian Locomotor Activity Rhythm

To quantify the circadian locomotor activity rhythm, the running wheel revolutions were recorded by a magnetic circuit, which was fixed vertically on the axis of the running wheel outside the cage. The revolutions (locomotor activity) were captured in 1 min intervals by the ClockLab 3 data acquisition system software (Acquisition Version 3.208, Analysis Version 6.0.36). This is further described in Jud et al., 2005 [27] and Brenna et al., 2025 [30].

### 4.3. Light Pulses Application (Aschoff Type I Protocol)

The experimental design (Figure 2) was performed according to the Aschoff Type I protocol [17,18]. Both male and female mice across all genotypes were tested using a side-by-side design. Mice were allowed to entrain in a constant 12:12 light:dark cycle for 2 weeks. This was followed by their release into constant darkness (DD) for 2 weeks, switching off the light by using an automatic digital timer. Following the 2 weeks in DD, the light pulse (LP) at circadian time (CT) 10 was calculated for each mouse individually by analyzing their wheel-running activity in the ClockLab3 software. In essence, the last 10 days of wheel-running activity in DD were used to obtain the period length and prediction of activity at CT12 for the next day. These were used to calculate the light pulse at CT10 for each mouse, as described in Jud et al., 2005 [27]. The LPs were applied the next day in a separate cabinet. Each mouse received a 15 min LP (using 2 visible light spectrum neon light sources: Lumilux cool daylight, 1000 Lux, 18W, OSRAM, Munich, Germany) at their previously calculated time, followed by their transfer into the original cabinet, where they were allowed to run freely for another 2 weeks in DD. The procedure described above was repeated for administering the LPs at CT14 and CT22 (Figure 2). At the end of the last DD entrainment, the mice were transferred back into a 12:12 light:dark cycle. A recovery time of 2 weeks was allowed before they were sacrificed for tissue collection.

### 4.4. Analysis of Circadian Locomotor Activity Rhythm Parameters

Phase shift (i.e., phase resetting) in DD following each LP, as well as other circadian locomotor activity rhythm parameters (e.g., actograms, period length, amplitude, total locomotor activity count, and relative power of phase (FFT)) were evaluated using ClockLab analysis (Actimetrics, Lafayette, IN, USA) software version 6.0.36.

To calculate the phase shift following each light pulse, a line of best fit was set through 10 consecutive activity onsets before the LP was applied. A second line of best fit was set through 10 activity onsets after the LP, excluding the first two days after the LP (transition phase). The difference between the two lines of best fit at the onset of activity during the day of the LP is considered the phase shift value.

All other circadian locomotor activity parameters were obtained using the ClockLab3 software, by setting the 2 lines of best fit through 10 activity onsets as described above.

### 4.5. Tissue Collections and Immunohistochemistry

Mice were perfused at ZT12 with 4% paraformaldehyde (PFA). Brains were isolated, with particular care taken not to damage the optical nerve area and subsequently the SCN. Brains were held overnight in 4% PFA before being cryoprotected in 30% sucrose for 2 days. SCN cryosections (40 µm) from each brain were placed in 24-well plates. Sections were washed for 20 min in 1× PBS buffer, followed by three 10 min washes in 1× PBS/0.2% Triton X-100 buffer. The sections were then treated for 15 min in 1× PBS/1% Triton X-100m buffer, before being washed 3 times with 1× PBS/0.2% Triton X-100. Sections were then blocked for 3 h at room temperature in 1× PBS/0.2% Triton X-100/10% Donkey Serum (Sigma, St. Louis, MI, USA, Catalog Number D9663). Following blocking, primary antibodies for PER1, GFAP, and NeuN (Table 1) were diluted in 1× PBS/0.2% Triton X-100/10% Donkey Serum, and sections were incubated at 4 °C for 2 nights. Following incubation, the sections were washed 5 times (10 min. each) with 1× PBS/0.2% Triton X-100 buffer before being incubated for 2 h at room temperature with the secondary antibodies (Table 1) diluted in 1× PBS/0.2% Triton X-100/10% Donkey Serum. This was then followed by 5 washes with 1× PBS/0.2% Triton X-100 and an overnight wash at 4 °C. All sections were stained with DAPI (Roche, Basel, Switzerland; 1:5000 in 1× PBS/0.2% Triton X-100) for 20 min. A final wash with 1× PBS/0.2% Triton X-100 was performed before being mounted on glass microscope slides. Fluorescent Z-stack images were taken using a confocal microscope (Leica TCS SP5, Leica Microsystems, Heerbrugg, Switzerland) using either the 20× or 63× magnification. All acquired images were processed in ImageJ, version 2.14.0/2.54f. The images of the SCN for each mouse genotype, acquired with either the 20× or 63× magnification, were analyzed by compressing the entire Z-stack. On the other hand, for the 3D analysis, no compression of the Z-stack was performed.

### 4.6. Statistical Analysis

GraphPad Prism software (version 10.3.1) was used for statistical analysis. Our aim was to compare cell-specific Per1KO against their respective Co lines (Ncre, GCre). Normality tests, using the Shapiro–Wilk test, were performed to assess the distribution of each group. We performed one-way Anova analysis, followed by Šídák’s multiple comparisons to investigate the effects of Per1 deletion against their control lines on period lengths, amplitudes, relative power of phase, and total wheel-running activity were analyzed via repeated measures two-way Anova, followed by Šídák’s multiple comparisons or Uncorrected Fisher’s LSD. The comparisons of these parameters on the WT mice were performed via paired t-tests. Data are presented as mean ± SEM and are considered significant when the *p*-value < 0.05.

The raw data appear in Appendix A.

## Figures and Tables

**Table 1:** Antibodies used for immunohistochemistry.

Antibody	Species	Company	Catalog Number	Lot Number	Dilution
Anti-mPER1 (residues 6-21)	Rabbit	Merck Millipore (Burlington, MA, USA)	AB2201	3480987	1:8000
Anti-GFAP	Goat	Abcam (Cambridge, UK)	AB53554	1046529-1	1:1000
Anti-NeuN	Chicken	Merck Millipore (Burlington, MA, USA)	ABN91	4049831	1:2000
Alexa Fluor 647-AffiniPure Donkey Anti-Chicken IgG (H + L)	Donkey	Jackson Immunoresearch (West Grove, PA, USA)	703-605-155	134612	1:1000
Alexa Fluor 568 Donkey Anti-Goat IgG (H + L)	Donkey	Abcam (Cambridge, UK)	Ab175704	GR3278446-1	1:1000
Alexa Fluor 488-AffiniPure Donkey Anti-Rabbit IgG (H + L)	Donkey	Jackson Immunoresearch (West Grove, PA, USA)	711-545-152	132876	1:1000

## Data Availability

The original contributions presented in this study are included in the article. Further inquiries can be directed to the corresponding author.

## Appendix A

**Raw Data**

**Figure 3B**

**Phase Shift—LP CT10**

**Table A1:** Raw data of phase responses in control (wt, NCo, GCo) and *Per1* knock-out animals (*Per1*NKo, *Per1*GKo) following a light pulse (LP) at CT10, corresponding to Figure 3B (left panel). Independent measures throughout light pulse conditions of both male and female C57BL/6 mice across all genotypes.

WT	NCo	*Per1*NKO	GCo	*Per1*GKo
12	7	−4	0	0
−2	−23	−18	−26	21
−12	−16	19	−28	−10
−12	23	17	−14	−9
28	32	−2	7	7
10	0	−30	−17	−9
7	10	6	6	−18
15	−3	15	10	−12
12	7	−18	−11	−3
−8	−14	−13	−20	3
	−10	3		
	−5	−9		

**Phase Shift—LP CT14**

**Table A2:** Raw data of phase responses in control (wt, NCo, GCo) and *Per1* knock-out animals (*Per1*NKo, *Per1*GKo) following a light pulse (LP) at CT14, corresponding to Figure 3B (middle panel). Independent measures throughout light pulse conditions of both male and female C57BL/6 mice across all genotypes.

WT	NCo	*Per1*NKO	GCo	*Per1*GKo
−147	−108	−115	−138	−127
−138	−115	−129	−120	−85
−70	−74	−120	−110	−172
−99	−60	−66	−113	−90
−108	−42	−71	−144	−108
−76	−82	−117	−128	−143
−143	−62	−90	−78	−174
−104	−128	−122	−99	−145
−138	−94	−111	−146	−118
−148	−82	−122	−211	−88
	−131	−83		
	−126	−146		

**Phase Shift—LP CT22**

**Table A3:** Raw data of phase responses in control (wt, NCo, GCo) and *Per1* knock-out animals (*Per1*NKo, *Per1*GKo) following a light pulse (LP) at CT22, corresponding to Figure 3B (right panel). Independent measures throughout light pulse conditions of both male and female C57BL/6 mice across all genotypes.

WT	NCo	*Per1*NKO	GCo	*Per1*GKo
39	7	39	50	25
30	34	25	65	39
40	28	−18	28	30
44	30	−6	39	55
58	25	9	30	21
19	32	−39	19	25
45	18	30	0	−24
39	16	1	51	71
28	74	21	14	10
25	20	32	66	16
	87	37		
	41	23		
	69	−15		
	66	48		
	73	67		
	58	85		
	25			
	45			
	30			
	63			

**Figure 4A—Period Length—Before and After LP CT10**

**Table A4:** Raw data of the period length in control (wt, NCo, GCo) and *Per1* knock-out animals (*Per1*NKo, *Per1*GKo) before and after a light pulse (LP) at CT10, corresponding to Figure 4A. Independent measures throughout light pulse conditions of both male and female C57BL/6 mice across all genotypes.

WT−	WT+	NCo−	NCo+	*Per1*NKO−	*Per1*NKO+	GCo−	GCo+	*Per1*GKO−	*Per1*GKO+
23.52	23.52	23.72	23.77	22.63	22.49	23.7	23.67	23.7	23.64
23.52	23.47	23.32	23.25	22.71	22.91	24.01	24.02	23.33	23.51
23.69	23.9	23.43	23.41	23.04	23.13	23.88	23.81	23.52	23.48
23.46	23.48	23.42	22.93	23.85	23.72	23.61	23.69	23.61	23.57
23.54	23.65	23.47	23.4	23.54	23.55	23.73	23.83	23.55	23.59
23.51	23.41	23.31	23.27	23.39	23.37	23.6	23.65	23.21	23.3
23.52	23.53	23.63	23.62	23.02	23.05	23.37	23.28	23.56	23.65
23.89	23.81	23.48	23.53	22.91	22.32	23.5	23.69	23.33	23.34
23.84	23.75	23.38	23.42	22.74	22.83	23.71	23.41	23.45	23.42
23.64	23.55	23.55	23.22	22.92	22.91	23.58	23.61	24.02	24.13
		23.57	23.62	22.98	23.17				
		23.51	23.43	23.77	23.71				

**Figure 4B—Period Length—Before and After LP CT14**

**Table A5:** Raw data of the period length in control (wt, NCo, GCo) and *Per1* knock-out animals (*Per1*NKo, *Per1*GKo) before and after a light pulse (LP) at CT14, corresponding to Figure 4B. Independent measures throughout light pulse conditions of both male and female C57BL/6 mice across all genotypes.

WT−	WT+	NCo−	NCo+	*Per1*NKo−	*Per1*NKo+	GCo−	GCo+	*Per1*GKO−	*Per1*GKo+
23.54	23.53	23.4	23.4	22.98	22.99	23.71	23.73	23.77	23.75
23.64	23.58	23.36	23.35	22.84	22.76	24.05	24.02	23.57	23.38
23.91	23.85	23.66	23.5	23.32	23.2	23.83	23.88	23.47	23.46
23.67	23.49	23.31	23.37	23.57	23.77	23.78	23.72	23.63	23.62
23.62	23.56	23.5	23.5	23.33	23.51	23.61	23.58	23.54	23.48
23.68	23.61	23.24	23.27	23.36	23.34	23.92	23.92	23.35	23.34
23.72	23.55	23.54	23.1	22.5	22.83	23.46	23.42	23.83	23.55
23.97	23.98	23.47	23.51	22.88	22.91	23.54	23.56	23.7	23.74
23.77	23.85	23.48	23.47	23.47	23.59	23.65	23.71	23.51	23.5
23.63	23.65	23.43	23.68	22.54	22.83	23.53	23.66	24.1	24.07
		23.34	23.38	22.82	22.86				
		23.35	23.47	23.77	23.77				

**Figure 4C—Period Length—Before and After LP CT22**

**Table A6:** Raw data of the period length in control (wt, NCo, GCo) and *Per1* knock-out animals (*Per1*NKo, *Per1*GKo) before and after a light pulse (LP) at CT22, corresponding to Figure 4C. Independent measures throughout light pulse conditions of both male and female C57BL/6 mice across all genotypes.

WT−	WT+	NCo−	NCo+	*Per1*NKO−	*Per1*NKO+	GCo−	GCo+	*Per1*GKO−	*Per1*GKO+
23.85	23.56	23.59	23.51	22.94	22.51	23.78	23.71	23.92	23.79
23.81	23.79	23.54	23.56	23.56	22.94	24.08	24	23.69	23.58
24.01	23.84	23.67	23.67	23.13	22.75	23.94	23.82	23.53	23.37
23.91	23.63	23.49	23.39	23.46	23.41	23.87	23.77	23.79	23.6
23.67	23.63	23.68	23.28	23.6	23.4	23.76	23.68	23.67	23.48
23.68	23.72	23.81	23.67	23.97	23.48	23.99	23.76	23.63	23.23
23.89	23.7	23.6	23.27	23.49	23.3	23.79	23.59	23.69	23.48
24.06	23.93	23.56	23.5	23.47	23.41	23.88	23.46	23.68	23.38
23.93	23.77	23.39	23.27	23.22	23.02	23.94	23.55	23.88	23.49
23.83	23.66	23.65	23.46	22.93	22.57	23.75	23.71	24.11	23.99
		23.87	23.25	22.76	22.72				
		23.81	23.64	22.95	22.49				
		23.54	23.4	23.54	23.42				
		23.49	23.44	23.41	22.89				
		23.56	23.37	23.07	22.81				
		23.58	23.3	23.91	23.67				
		23.73	23.56						
		23.42	23.39						
		23.64	23.34						
		23.68	23.55						

**Figure 5A—Amplitude—Before and After LP CT10**

**Table A7:** Raw data of the amplitudes in wt animals before and after a light pulse (LP) at CT10, corresponding to Figure 5A (left panel). Independent measures throughout light pulse conditions of both male and female C57BL/6 mice across all genotypes.

WT−	WT+
1629.2	1673.6
596.2	484.83
1630.28	1651.32
1291.7	1170.19
1708.38	1881.72
1462.28	1744.38
1477	1625.51
1394.99	1103.68
1476.33	1515.13
1412.77	1442.94

**Table A8:** Raw data of the amplitudes in control (Nco) and *Per1*NKo animals before and after a light pulse (LP) at CT10, corresponding to Figure 5A (middle panel). Independent measures throughout light pulse conditions of both male and female C57BL/6 mice across all genotypes.

NCo−	NCo+	*Per1*NKO−	*Per1*NKO+
1566.23	1630.78	1022.9	914.22
1367.67	1368.14	679.31	631.27
1525.49	1519.59	1533.5	1309.38
1895.58	1911.1	1269.11	1179.53
1394.79	1565.67	1256.69	1470.32
1588	1784.54	911.89	784.27
1150.29	1185.78	1092.38	1123.25
879.61	1120.03	1180.42	1047.43
1737.62	1769.17	1187.93	1174.27
1405.35	1654.24	1305.9	1262.47
1066.06	1186.45	961.34	1060.84
1604.57	1771.29	1110.39	1303.44

**Table A9:** Raw data of the amplitudes in control (Gco) and *Per1*GKo animals before and after a light pulse (LP) at CT10, corresponding to Figure 5A (right panel). Independent measures throughout light pulse conditions of both male and female C57BL/6 mice across all genotypes.

GCo−	GCo+	*Per1*GKO−	*Per1*GKO+
1420.29	1181	968.53	1097.58
1253.68	1146.13	1232.85	920.71
1043.8	887.32	1291.7	1170.19
934.56	910.72	963.98	893.04
1346.69	1362.21	1464.79	1534.84
1029.59	1106.36	1274.09	1088.74
1421.2	1427.64	1051.08	970.53
1320.21	1469.69	1006.68	907.49
1172.18	1173.28	1609.85	1195.26
1147.91	1064.07	639.8	1062.97

**Figure 5B—Amplitude—Before and After LP CT14**

**Table A10:** Raw data of the amplitude in wt animals before and after a light pulse (LP) at CT14, corresponding to Figure 5B (left panel). Independent measures throughout light pulse conditions of both male and female C57BL/6 mice across all genotypes.

WT−	WT+
1700.28	1731
1025.83	679.8
1793.12	1658.44
1561.66	1578.08
1716.78	1780.54
1443.52	1616.54
1046.23	1413.56
1347.69	1433.07
1385.71	1511.1
1288.71	1491.23

**Table A11:** Raw data of the amplitudes in control (Nco) and *Per1*NKo animals before and after a light pulse (LP) at CT14, corresponding to Figure 5B (middle panel). Independent measures throughout light pulse conditions of both male and female C57BL/6 mice across all genotypes.

NCo−	NCo+	*Per1*NKO−	*Per1*NKO+
1675.76	1615.08	1234.96	1048.78
1494.18	1429.02	831.3	664.68
1676.09	1520.32	1414.74	1535.74
1322.45	1534.78	1452.98	1378.7
1438.15	1531.97	1205.68	1464.62
1769.83	1535.75	1083.32	1289.99
1408.81	1089.65	1057.86	1185.5
1281.54	1504.97	1066.39	1207.54
1320.81	1573.63	1015.99	1270.6
1580.79	1650.43	1246.28	1575.57
1794.14	1747.38	1156.43	1331.06
1154.33	1057.54	1063.21	855.05

**Table A12:** Raw data of the amplitudes in control (Gco) and *Per1*GKo animals before and after a light pulse (LP) at CT14, corresponding to Figure 5B (right panel). Independent measures throughout light pulse conditions of both male and female C57BL/6 mice across all genotypes.

GCO−	GCo+	*Per1*GKO−	*Per1*GKO+
1420.29	1181	968.53	1097.58
1253.68	1146.13	1232.85	920.71
1043.8	887.32	1291.7	1170.19
934.56	910.72	963.98	893.04
1346.69	1362.21	1464.79	1534.84
1029.59	1106.36	1274.09	1088.74
1421.2	1427.64	1051.08	970.53
1320.21	1469.69	1006.68	907.49
1172.18	1173.28	1609.85	1195.26
1147.91	1064.07	639.8	1062.97

**Figure 5C—Amplitude—Before and After LP CT22**

**Table A13:** Raw data of the amplitude in wt animals before and after a light pulse (LP) at CT22, corresponding to Figure 5C (left panel). Independent measures throughout light pulse conditions of both male and female C57BL/6 mice across all genotypes.

WT−	WT+
1073.47	1062.06
1670.84	1784.74
1476.21	1601.83
1588.3	1832.59
1135.89	1156.47
1479.6	1327.47
1116.26	961.53
1339.08	1521.42
1587.32	1640.92
1238.91	1340.14

**Table A14:** Raw data of the amplitudes in control (Nco) and *Per1*NKo animals before and after a light pulse (LP) at CT22, corresponding to Figure 5C (middle panel). Independent measures throughout light pulse conditions of both male and female C57BL/6 mice across all genotypes.

NCo−	NCO+	*Per1*NKO−	*Per1*NKO+
1481.56	1529.37	1227.28	1189.36
1624.38	1409.12	1023	850.79
1861.74	1676.92	1244.99	1461.47
1448.02	1667.55	1381.32	1438.58
1255.35	1497.07	727.42	1241.72
1375.49	1412.21	1274.48	1178.82
959.37	1068.34	691.59	936.2
1819.02	1752.56	971.88	1207.46
997.71	961.52	1174.01	1162.88
1150.3	1556.34	1051.52	1034.11
1033.47	1434.64	1496.9	1440.03
1427.15	1426.25	894.46	984.58
1296.44	1384.99	1364.76	1330.06
961.63	1306.92	1176.71	1194.95
1586.6	2086.73	989.71	1076.45
1902.79	1823.62	1450.16	1423.16
1261.72	1206.29		
1539.68	1891.81		
1927.48	1987.97		
1508.26	1490.64		

**Table A15:** Raw data of the amplitudes in control (Gco) and *Per1*GKo animals before and after a light pulse (LP) at CT22, corresponding to Figure 5C (right panel). Independent measures throughout light pulse conditions of both male and female C57BL/6 mice across all genotypes.

GCo−	GCo+	*Per1*GKO−	*Per1*GKO+
1518.92	1282.65	1070.15	785.52
1405.78	1383.96	1371.63	1299.42
1252.48	1127.76	1319.08	1325.03
1522.85	1714.34	1139.27	1006.39
952.73	1042.12	1631.8	1721.83
1175.99	1458.97	966.08	1253.66
1280.26	1409.2	1021.15	818.92
1136.51	1507.97	1235.66	1200.19
1133.49	794.41	1098.63	1591.71
1102.36	1276.25	1208.2	780.88

**Figure 6A—Relative Power of Phase—Before and After LP CT10**

**Table A16:** Raw data of the relative power of phase in wt animals before and after a light pulse (LP) at CT10, corresponding to Figure 6A (left panel). Independent measures throughout light pulse conditions of both male and female C57BL/6 mice across all genotypes.

WT−	WT+
0.0115	0.0119
0.0032	0.0022
0.0104	0.0105
0.01	0.0089
0.0121	0.0139
0.0114	0.0114
0.008	0.0081
0.0132	0.011
0.0087	0.0083
0.0106	0.0111

**Table A17:** Raw data of the relative power of phase in control (Nco) and *Per1*NKo animals before and after a light pulse (LP) at CT10, corresponding to Figure 6A (middle panel). Independent measures throughout light pulse conditions of both male and female C57BL/6 mice across all genotypes.

NCo−	NCo+	*Per1*NKO−	*Per1*NKO+
0.0102	0.0098	0.0049	0.0049
0.0095	0.0093	0.0041	0.0033
0.0094	0.0075	0.0076	0.0068
0.0149	0.0155	0.0052	0.0044
0.0097	0.0092	0.0079	0.0076
0.0127	0.0129	0.0044	0.0045
0.0156	0.0144	0.0075	0.007
0.007	0.008	0.0075	0.0072
0.0094	0.0112	0.0068	0.0062
0.0119	0.012	0.0068	0.0052
0.0126	0.0134	0.005	0.0046
0.0177	0.0152	0.0068	0.0065

**Table A18:** Raw data of the relative power of phase in control (Gco) and *Per1*GKo animals before and after a light pulse (LP) at CT10, corresponding to Figure 6A (right panel). Independent measures throughout light pulse conditions of both male and female C57BL/6 mice across all genotypes.

GCo−	GCo+	*Per1*GKO−	*Per1*GKO+
0.0098	0.0093	0.0053	0.0055
0.0082	0.008	0.0077	0.0065
0.007	0.0053	0.007	0.0061
0.01	0.0095	0.0058	0.0055
0.0055	0.0054	0.0088	0.0083
0.0077	0.0088	0.01	0.0092
0.0145	0.0141	0.0111	0.0102
0.011	0.0107	0.0081	0.0064
0.0131	0.0139	0.0109	0.0101
0.0112	0.0105	0.006	0.0089

**Figure 6B—Relative Power of Phase—Before and After LP CT14**

**Table A19:** Raw data of the relative power of phase in wt animals before and after a light pulse (LP) at CT14, corresponding to Figure 6B (left panel). Independent measures throughout light pulse conditions of both male and female C57BL/6 mice across all genotypes.

WT−	WT+
0.0121	0.0123
0.005	0.0033
0.0116	0.0103
0.0104	0.0104
0.0118	0.0121
0.0124	0.0129
0.0062	0.0069
0.0157	0.0145
0.0101	0.0091
0.0107	0.011

**Table A20:** Raw data of the relative power of phase in control (Nco) and *Per1*NKo animals before and after a light pulse (LP) at CT14, corresponding to Figure 6B (middle panel). Independent measures throughout light pulse conditions of both male and female C57BL/6 mice across all genotypes.

NCo−	NCo+	*Per1*NKO−	*Per1*NKO+
0.0108	0.0107	0.0053	0.0046
0.0095	0.0095	0.0051	0.0042
0.0099	0.0094	0.007	0.0075
0.0107	0.0102	0.0078	0.0094
0.0138	0.0127	0.0068	0.0056
0.0105	0.0112	0.0084	0.0078
0.0118	0.0086	0.0085	0.0073
0.0099	0.0104	0.0076	0.0069
0.0119	0.0145	0.0079	0.0075
0.0118	0.0124	0.0067	0.0075
0.0146	0.0125	0.006	0.007
0.0117	0.0099	0.0066	0.0077

**Table A21:** Raw data of the relative power of phase in control (Gco) and *Per1*GKo animals before and after a light pulse (LP) at CT14, corresponding to Figure 6B (right panel). Independent measures throughout light pulse conditions of both male and female C57BL/6 mice across all genotypes.

GCo−	GCo+	*Per1*GKO−	*Per1*GKO+
0.0093	0.0095	0.0046	0.0055
0.0094	0.0084	0.0073	0.0072
0.0086	0.0077	0.0077	0.0073
0.0122	0.011	0.0068	0.0061
0.0062	0.0059	0.0088	0.0097
0.0119	0.0135	0.0091	0.0098
0.014	0.0152	0.0089	0.011
0.0089	0.0077	0.0079	0.0083
0.0122	0.0119	0.0101	0.0089
0.0079	0.014	0.0118	0.012

**Figure 6C—Relative Power of Phase—Before and After LP CT22**

**Table A22:** Raw data of the relative power of phase in wt animals before and after a light pulse (LP) at CT22, corresponding to Figure 6C (left panel). Independent measures throughout light pulse conditions of both male and female C57BL/6 mice across all genotypes.

WT−	WT+
0.0048	0.0049
0.0114	0.0116
0.0097	0.0103
0.011	0.0119
0.0069	0.0073
0.0121	0.0122
0.0133	0.0157
0.0116	0.0111
0.0062	0.0062
0.0106	0.0106

**Table A23:** Raw data of the relative power of phase in control (Nco) and *Per1*NKo animals before and after a light pulse (LP) at CT22, corresponding to Figure 6C (middle panel). Independent measures throughout light pulse conditions of both male and female C57BL/6 mice across all genotypes.

NCo−	NCo+	*Per1*NKO−	*Per1*NKO+
0.0106	0.0113	0.0058	0.0055
0.0129	0.0106	0.0054	0.0049
0.0118	0.0101	0.0091	0.0069
0.0121	0.0123	0.0095	0.0083
0.0082	0.0115	0.0058	0.0068
0.0065	0.0061	0.0073	0.0083
0.0125	0.0166	0.004	0.0048
0.0168	0.0173	0.0115	0.0122
0.0125	0.0136	0.0075	0.0086
0.0133	0.011	0.0071	0.0079
0.0059	0.0064	0.0069	0.0068
0.0143	0.0124	0.0085	0.0076
0.0105	0.0098	0.0049	0.0053
0.0132	0.0166	0.0068	0.0072
0.0108	0.0126	0.0092	0.0076
0.0143	0.0132	0.0136	0.0166
0.0144	0.0159		
0.0127	0.0138		
0.0137	0.0185		
0.018	0.0169		

**Table A24:** Raw data of the relative power of phase in control (Gco) and *Per1*GKo animals before and after a light pulse (LP) at CT22, corresponding to Figure 6C (right panel). Independent measures throughout light pulse conditions of both male and female C57BL/6 mice across all genotypes.

GCo−	GCo+	*Per1*GKO−	*Per1*GKO+
0.0103	0.0098	0.0071	0.0046
0.01	0.0101	0.0079	0.007
0.008	0.0082	0.0085	0.0078
0.0102	0.0128	0.0071	0.0064
0.0058	0.0061	0.0108	0.0089
0.0105	0.0116	0.0087	0.0091
0.0096	0.0122	0.0103	0.0092
0.0139	0.0145	0.0096	0.0077
0.0109	0.0126	0.008	0.0106
0.0125	0.0084	0.0099	0.0072

**Figure 7A—Wheel Revolutions—Before and After LP CT10**

**Table A25:** Raw data of the wheel revolutions of in wt animals before and after a light pulse (LP) at CT10, corresponding to Figure 7A (left panel). Independent measures throughout light pulse conditions of both male and female C57BL/6 mice across all genotypes.

WT−	WT+
244,734	240,866
46,792	32,568
237,729	236,200
217,243	188,537
454,149	520,440
249,152	250,230
146,302	136,451
389,652	342,849
312,362	262,004
341,940	351,375

**Table A26:** Raw data of wheel revolutions in control (Nco) and *Per1*NKo animals before and after a light pulse (LP) at CT10, corresponding to Figure 7A (middle panel). Independent measures throughout light pulse conditions of both male and female C57BL/6 mice across all genotypes.

NCo−	NCo+	*Per1*NKO−	*Per1*NKO+
218,791	205,467	111,072	126,750
252,929	217,789	119,075	102,913
342,744	262,696	322,705	276,080
482,338	572,646	204,308	149,835
313,478	355,716	193,381	180,136
330,132	295,652	198,051	206,006
637,773	610,999	203,996	150,433
234,887	261,367	454,497	374,048
384,940	441,118	423,743	378,609
339,336	353,439	315,264	322,637
345,426	352,075	210,797	291,156
249,229	261,962	322,166	319,490

**Table A27:** Raw data of the wheel revolutions in control (Gco) and *Per1*GKo animals before and after a light pulse (LP) at CT10, corresponding to Figure 7A (right panel). Independent measures throughout light pulse conditions of both male and female C57BL/6 mice across all genotypes.

GCo−	GCo+	*Per1*GKO−	*Per1*GKO+
296,336	255,942	105,882	107,544
229,251	214,979	144,644	130,187
185,580	134,745	179,670	168,521
195,696	178,052	204,501	169,353
159,084	144,890	290,940	275,816
224,838	199,862	416,302	411,713
448,138	433,318	336,436	337,461
318,614	339,488	314,908	242,631
426,093	410,714	322,923	266,675
331,914	299,191	142,074	283,565

**Figure 7B—Wheel Revolutions—Before and After LP CT14**

**Table A28:** Raw data of the wheel revolutions of in wt animals before and after a light pulse (LP) at CT14, corresponding to Figure 7B (left panel). Independent measures throughout light pulse conditions of both male and female C57BL/6 mice across all genotypes.

WT−	WT+
240,410	254,367
82,311	49,743
285,137	242,927
215,179	227,147
376,859	462,758
278,298	270,004
129,260	134,719
417,326	380,632
352,962	318,603
350,868	350,632

**Table A29:** Raw data of wheel revolutions in control (Nco) and *Per1*NKo animals before and after a light pulse (LP) at CT14, corresponding to Figure 7B (middle panel). Independent measures throughout light pulse conditions of both male and female C57BL/6 mice across all genotypes.

NCo−	NCo+	*Per1*NKO−	*Per1*NKO+
251,290	231,417	156,910	115,182
280,599	265,181	118,707	115,494
342,037	339,369	262,451	321,276
254,022	320,806	289,847	310,230
533,059	446,784	178,316	207,862
292,090	300,417	231,630	200,888
378,389	550,061	386,629	432,737
233,259	228,652	178,930	173,431
613,617	604,298	200,160	222,907
461,801	545,264	398,227	479,472
290,516	298,258	374,886	409,356
330,929	339,254	360,278	320,689

**Table A30:** Raw data of the wheel revolutions in control (Gco) and *Per1*GKo animals before and after a light pulse (LP) at CT14, corresponding to Figure 7B (right panel). Independent measures throughout light pulse conditions of both male and female C57BL/6 mice across all genotypes.

GCo−	GCo+	*Per1*GKO−	*Per1*GKO+
262,020	285,084	96,965	107,620
306,433	230,807	157,855	134,910
175,325	191,835	170,113	174,704
242,560	195,258	194,854	207,609
148,531	163,719	241,263	285,885
362,979	386,668	356,582	406,299
397,118	448,138	314,382	335,690
239,686	227,843	246,404	334,522
348,745	349,613	364,804	390,672
208,570	438,812	250,504	159,382

**Figure 7C—Wheel Revolutions—Before and After LP CT22**

**Table A31:** Raw data of the wheel revolutions of in wt animals before and after a light pulse (LP) at CT22, corresponding to Figure 7C (left panel). Independent measures throughout light pulse conditions of both male and female C57BL/6 mice across all genotypes.

WT−	WT+
92,557	85,671
262,836	277,889
200,624	213,688
313,694	421,147
175,633	214,912
268,751	267,469
136,337	111,548
387,280	413,122
344,158	369,719
327,321	361,340

**Table A32:** Raw data of wheel revolutions in control (Nco) and Per1NKo animals before and after a light pulse (LP) at CT22, corresponding to Figure 7C (middle panel). Independent measures throughout light pulse conditions of both male and female C57BL/6 mice across all genotypes.

NCo−	NCo+	P1NKO−	P1NKO+
239,390	234,918	160,751	140,374
312,436	260,558	129,961	113,624
354,334	342,854	219,735	256,441
317,156	337,413	257,202	282,733
169,070	244,920	133,229	168,874
128,860	93,636	146,894	230,736
416,713	502,006	176,013	181,463
519,500	593,352	272,844	290,042
292,209	296,689	165,029	228,320
371,353	386,950	376,412	397,206
193,241	229,756	268,641	378,746
431,143	431,138	358,247	421,444
361,284	406,581	208,696	206,332
256,446	338,402	317,752	306,705
252,999	339,745	343,732	408,438
338,345	341,841	261,867	268,968
366,863	317,026		
219,944	252,265		
493,689	660,953		
449,941	495,378		

**Table A33:** Raw data of the wheel revolutions in control (Gco) and Per1GKo animals before and after a light pulse (LP) at CT22, corresponding to Figure 7C (right panel). Independent measures throughout light pulse conditions of both male and female C57BL/6 mice across all genotypes.

GCo−	GCo+	P1GKO−	P1GKO+
256,610	234,705	145,045	97,931
308,817	275,293	179,235	160,647
246,088	182,287	179,498	170,569
212,203	247,235	190,443	182,077
194,307	148,037	313,510	268,019
337,609	387,877	277,649	374,044
326,693	427,769	318,375	316,719
343,640	387,117	275,483	270,982
430,180	268,466	207,919	339,585
319,850	362,163	335,811	180,523
